# Acute Aortic Syndrome: From Risk Factors to Hospital Burden and Healthcare Resource Utilization

**DOI:** 10.3390/clinpract16070121

**Published:** 2026-06-27

**Authors:** Cosmin Marian Banceu, Diana Mariana Banceu, Marius Mihai Harpa, Daiana Cristutiu, Mihai Calinescu, Horatiu Suciu

**Affiliations:** 1 Department ME 2, George Emil Palade University of Medicine, Pharmacy, Science, and Technology of Targu Mures, 540139 Targu Mures, Romania; cosmin.banceu@umfst.ro; 2 Emergency Institute for Cardiovascular Diseases and Transplantation Targu Mures, 540136 Targu Mures, Romania; 3 Organizing Institution for Doctoral University Studies, West University of Timisoara, 300223 Timisoara, Romania; 4 Department of Phycology, George Emil Palade University of Medicine, Pharmacy, Science and Technology, 540142 Targu-Mures, Romania; 5 Department M3, George Emil Palade University of Medicine, Pharmacy, Science, and Technology of Targu Mures, 540139 Targu Mures, Romania; 6 Institution Organising Doctoral Study Programmes, George Emil Palade University of Medicine, Pharmacy, Science, and Technology of Targu Mures, 540139 Targu Mures, Romania

**Keywords:** acute aortic syndrome, aortic dissection, risk factors, prolonged hospitalization, outcome

## Abstract

Acute aortic syndrome (AAS) comprises acute aortic dissection, intramural haematoma, penetrating atherosclerotic ulcer, and limited intimal tear, conditions that require rapid recognition because mortality and resource use are strongly influenced by time to diagnosis, anatomical extent, malperfusion, and the need for emergency surgical or endovascular intervention. This revised narrative review synthesizes contemporary evidence on clinical, genetic, environmental, and health-system determinants of prolonged hospitalisation, intensive care unit (ICU) utilisation, bed occupancy, and costs in patients with AAS. Beyond summarising established risk factors, the review adds a resource-oriented framework that links hypertension, advanced age, female sex, smoking-related comorbidity, hereditary aortopathies, haemodynamic instability, malperfusion, delayed diagnosis, operative complexity, and postoperative complications to measurable downstream outcomes such as ICU length of stay, total hospital length of stay, reoperation, readmission, and longitudinal imaging surveillance. We searched PubMed/MEDLINE, Scopus, Web of Science, and Google Scholar for relevant studies, registries, guideline documents, and cost analyses published between January 2000 and May 2026, with particular emphasis on studies from the last five years. The review was not designed as a meta-analysis; therefore, effect estimates are interpreted according to study design and generalisability. AAS imposes a disproportionate burden on hospital systems because high-risk patients often require advanced imaging, prolonged haemodynamic monitoring, complex open or endovascular repair, ICU care, and lifelong follow-up. Earlier diagnosis, structured risk stratification, targeted genetic evaluation, aggressive control of modifiable risk factors, and system-level pathways such as dedicated aortic networks may shorten hospital stay and reduce avoidable costs.

## 1. Introduction

Acute aortic syndrome (AAS) is an umbrella term for a group of time-critical aortic emergencies that share overlapping clinical presentations but differ in wall pathology, natural history, and treatment thresholds. The core entities are acute aortic dissection (AAD), intramural haematoma (IMH), penetrating atherosclerotic ulcer (PAU), and limited intimal tear, also described in some literature as intimal disruption.

AAD results from an intimal tear that permits blood to enter the medial layer and create a false lumen. IMH is characterised by haemorrhage within the aortic media without a clearly demonstrable intimal tear on initial imaging. PAU refers to atherosclerotic ulceration penetrating through the internal elastic lamina into the media. Limited intimal tear denotes a focal intimal disruption that may be radiologically subtle and can be missed if multiplanar imaging review is not performed.

The pathophysiological continuum among these entities is clinically important. IMH can progress to classic dissection or rupture, PAU can precipitate IMH or pseudoaneurysm formation, and limited intimal tear may mimic early dissection. From a prognostic and resource-use perspective, involvement of the ascending aorta, branch-vessel malperfusion, haemodynamic instability, organ dysfunction, frailty, and the need for extensive arch or descending aortic repair are more directly linked to ICU utilisation and total hospital stay than the diagnostic label alone.

Individuals with IMH exhibit comparable risk factors to those with AAD; however, IMH generally manifests in older adults, frequently within the eighth decade of life. IMH constitutes between 5% and 25% of AAS cases, with an estimated incidence of 1.2 per 100,000 patient-years. PAU accounts for 2–7% of AAS cases, exhibiting an incidence rate of 2.1 per 100,000 person-years. PAU is closely linked to severe diffuse atherosclerotic disease and often presents as numerous ulcerative lesions along the thoracic or abdominal aorta.

Comorbid diseases are common, including systemic hypertension and coronary artery disease; significantly, up to 68% of individuals demonstrate chronic obstructive pulmonary disease (COPD). Additionally, concurrent aortic aneurysms are noted in 42–61% of instances [1].

## 2. Methods: Narrative Review Strategy

This article is a narrative review. It was not registered as a systematic review, and no formal meta-analysis or pooled risk estimate was planned. To make the review process transparent, we added a concise description of the search strategy, evidence selection, and interpretive approach.

Literature searches were performed in PubMed/MEDLINE, Scopus, Web of Science, and Google Scholar for publications from January 2000 to May 2026. Search terms included combinations of “acute aortic syndrome”, “acute aortic dissection”, “intramural haematoma”, “penetrating atherosclerotic ulcer”, “limited intimal tear”, “risk factors”, “hospital length of stay”, “ICU length of stay”, “bed occupancy”, “healthcare resource use”, “cost”, “elderly”, “female sex”, “hypertension”, “smoking”, “genetic aortopathy”, “Marfan syndrome”, “Loeys–Dietz syndrome”, “malperfusion”, and “aortic network” (Table 1).

Eligible sources included contemporary guidelines, population-based cohorts, registry analyses, multicentre cohort studies, single-centre observational studies, cost-of-illness analyses, systematic reviews, and mechanistic reviews when they informed the link between risk factors and hospital resource utilisation. Priority was given to peer-reviewed studies published within the previous five years when available, while older landmark studies were retained when they provided foundational epidemiological or operative data.

Because study designs, endpoints, and healthcare systems were heterogeneous, quantitative claims are interpreted by study type and context rather than treated as pooled estimates. Where a finding is derived from a single-country administrative dataset, such as length-of-stay data in Japanese octogenarians, this limitation is explicitly stated.

## 3. Epidemiological Data on AAS

The existing knowledge about the epidemiology, etiopathogenesis, and prognostic factors of AAS is significantly inadequate [2,3,4]. Institutional datasets, including retrospective case series like the International Registry of Acute Aortic Dissection (IRAD) and case–control studies, are inherently vulnerable to ascertainment bias, mainly due to the poor capture of pre-hospital death. The epidemiology of AAS remains difficult to define because pre-hospital death, sudden unexplained death, and incomplete capture of fatal events can lead to underestimation of incidence and case fatality. Registry-based data such as IRAD provide important clinical detail, whereas population-based cohorts better capture incidence and pre-hospital mortality. These sources should therefore be interpreted as complementary rather than interchangeable. This constraint leads to a systematic underestimating of incidence and case fatality rates, hence undermining the internal validity of risk factor characterisation and outcome prediction models [5,6,7]. In contrast, recent population-based longitudinal cohort studies have provided more accurate estimates of real incidence and have clarified essential demographic and clinical risk factors [8,9,10]. Data from Swedish and British registries show that the age- and sex-adjusted annual incidence of AAD is between 6.0 and 7.2 cases per 100,000 person-years [8,11]. The annual incidence of acute aortic dissection is commonly reported in cases per 100,000 person-years. To avoid inconsistent epidemiological terminology, the revised manuscript uses “person-years” throughout when referring to population incidence rates. IMH and PAU are described as proportions of AAS presentations or as incidence estimates only when the original study design supports this expression. A longitudinal study in Olmsted County, Minnesota, reported an aggregate incidence of 7.7 per 100,000 person-years for all acute aortic pathologies, including CD, IMH, and PAU. In this cohort, CD represented 4.4 cases per 100,000 person-years, while PAU and IMH demonstrated lower frequencies [10]. Intimal disruption (ID) is frequently underdiagnosed and accounts for approximately 5% of presentations of AAS [12,13]. Stanford type A lesions are predominantly observed in cases of CD and ID, while Stanford type B lesions are more commonly linked to IMH and PAU [10]. Epidemiological analyses reveal a significant gender-specific disparity, with males showing an incidence nearly double that of females, alongside a pronounced age-dependent gradient in risk [10,11]. The average age at diagnosis differs by subtype, with the lowest for CD and the highest for PAU [10]. The mean age at presentation for CD is reported to range from 66 to 72 years, with female patients presenting at a significantly older age than male patients [5,8,10,11,14,15]. Recent guideline updates and contemporary cohort data reinforce the need for structured diagnostic pathways, rapid access to computed tomography angiography, blood pressure control, multidisciplinary decision-making, and early transfer to experienced aortic centres when ascending aortic involvement, malperfusion, rupture risk, or complicated type B disease is suspected.

Women and older adults remain particularly vulnerable to delayed diagnosis because they may present with atypical symptoms, less specific pain patterns, syncope, neurological manifestations, or more advanced physiological compromise. Diagnostic delay is not only a mortality issue; it also increases the probability of organ malperfusion, emergency surgery, ICU admission, and prolonged postoperative recovery.

Notwithstanding these advancements, consensus-based frameworks for risk stratification and evidence-based diagnostic thresholds are still insufficiently delineated. Moreover, algorithmic diagnostic methods for AAS are inconsistently applied across emergency care environments, leading to significant variability in clinical practice [16].

## 4. Risk Factors

Risk factors in AAS should be interpreted at two levels: first, as determinants of disease occurrence, and second, as determinants of clinical complexity once AAS has occurred. The latter is most relevant to prolonged hospitalisation. Hypertension, smoking, atherosclerosis, connective tissue disease, genetic aortopathy, advanced age, sex-related diagnostic delay, malperfusion, haemodynamic instability, renal dysfunction, prolonged operative time, and cardiopulmonary bypass duration influence resource use through different but converging pathways [17]. Other etiological factors include inflammatory vasculitis such as giant cell arteritis, infections like syphilis, mechanical trauma, and iatrogenic injuries resulting from endovascular procedures or previous aortic reconstructive surgeries (Table 2) [17,18].

Systemic arterial hypertension is the most prevalent modifiable risk factor and contributes to chronic medial degeneration, aneurysmal remodelling, and increased wall stress. In the acute setting, uncontrolled or unstable blood pressure complicates haemodynamic management, increases the probability of end-organ injury, and can prolong ICU monitoring. Long-term antihypertensive therapy and surveillance imaging further contribute to downstream healthcare costs. However, its prevalence at initial clinical presentation is lower in patients with type A (proximal) dissections compared to those with type B (distal) dissections (approximately 36% versus 70%, respectively). Factors linked to an increased risk of negative outcomes encompass chronic systemic hypertension, advanced age, enlarged aortic diameter, and the continued presence of a patent false lumen after dissection. Epidemiological evidence indicates that individuals with hypertension have more than a twofold increased risk of AAS compared to normotensive individuals, with around 54% of AAS cases linked to hypertension [9]. Landenhed et al. conducted a large-scale prospective analysis and found that 86% of patients who later developed aortic dissection had a documented history of hypertension, highlighting its significant pathophysiological association [9]. Despite the strong correlation, hypertension is not included as a significant predictive factor in the Aortic Dissection Detection Risk Score [19]. Antecedent hypertension is an independent and reliable clinical predictor for type A acute aortic dissection [20]. Consequently, stringent management of blood pressure is fundamental to preventive strategies. Nearly 50% of individuals with hypertension globally are unaware of their condition, underscoring the necessity for comprehensive educational interventions aimed at both patients and healthcare professionals [21]. Considering the relative rarity of AAS in contrast to the widespread occurrence of hypertension, it is essential to clarify the mechanistic foundations of this relationship. Recent developments in molecular genetics, vascular biology, and translational research may enhance risk stratification and predictive modelling, thereby supporting precision medicine strategies to reduce AAS-related morbidity and mortality. Hereditary aortopathies, including Marfan syndrome, Loeys–Dietz syndrome, and vascular Ehlers-Danlos syndrome, affect resource use by shifting disease onset to younger ages, increasing the probability of complex anatomy, requiring specialised surgical planning, and necessitating lifelong surveillance. Early genetic recognition can convert catastrophic emergency care into elective or planned care in selected patients, thereby reducing ICU dependence and reintervention risk.

Neurological manifestations, including syncope, may occur simultaneously with acute pain, indicating impaired cerebral or peripheral blood flow. This phenomenon is mainly due to impaired spinal and aortic outflow resulting from structural pathology of the aortic wall, which disrupts systemic haemodynamic and regional blood supply [22]. Ascending aortic dissection is linked to significant complications, such as myocardial ischaemia caused by coronary malperfusion, aortic regurgitation due to valvular involvement, and pericardial effusion resulting from haemorrhagic infiltration of the pericardial space.

Conditions, whether acquired or heritable, that undermine the structural integrity of the aortic wall significantly elevate the risk of spontaneous AAS [2,23,24]. The familial clustering of thoracic aortic disease is well established [25,26]. A documented family history of aortic dissection is a significant independent risk factor for thoracic aortic aneurysm and dissection, regardless of the presence of syndromic connective tissue disorders. The pathogenesis of AAS involves a complex interaction between genetic factors and environmental influences. Therefore, systematic imaging surveillance and molecular genetic testing are recommended for individuals with a family history of AAS or thoracic aortic aneurysm. Proactive risk stratification, aggressive management of contributing factors, and prophylactic aortic replacement in selected high-risk cohorts may reduce the incidence of catastrophic aortic events. Thoracic aortic aneurysm is a recognised risk factor for aortic dissection and rupture [2]. Current consensus guidelines support elective surgical intervention based mainly on absolute aortic diameter thresholds [2,3,27]. However, the effectiveness of these preventive strategies at the population level in reducing the burden of AAS is still unclear. Epidemiological trends indicate a steady increase in the prevalence of thoracic aortic aneurysms, accompanied by a rise in the rates of elective surgical repairs [6,28]. Simultaneously, procedural volumes for AAS management have shown a comparable increase [20,29].

Extensive epidemiological research has shown a notable correlation between fluoroquinolone exposure and a heightened occurrence of AAD and aortic aneurysm [30]. The administration of fluoroquinolones in patients with a predisposition to aortic pathology may lead to significant adverse effects [21]. Consequently, these agents should be avoided in patients with an increased risk for AAS when clinically suitable alternative antimicrobial therapies are accessible [31]. Mechanical stressors, such as traumatic injury and iatrogenic instrumentation, are significant risk factors for AAS, especially when coupled with underlying inflammatory processes or genetic predisposition [2,23,24,32]. Epidemiological data consistently demonstrate a male predominance, with younger patients often showing genetic predispositions [2,24]. Advanced age is associated with frailty, multimorbidity, renal vulnerability, pulmonary complications, delirium, and slower functional recovery. The Japanese study by Ohnuma et al. reported longer hospital stays in patients aged 80 years or older after type A AAD repair; however, this estimate should not be generalised uncritically to other health systems because discharge practices, rehabilitation pathways, case selection, and administrative reimbursement differ across countries.

Sex-related differences are clinically relevant but should be interpreted carefully. Female patients often present at an older age and may experience delayed recognition or more severe physiological compromise at admission. These factors can translate into higher acuity, longer recovery, and greater post-discharge resource utilisation, even when early postoperative mortality after surgical repair is not consistently different in all cohorts.

Systemic inflammatory vasculitis, including Takayasu arteritis and giant cell arteritis, increase the risk of aortic dissection during invasive procedures. Additionally, temporal analyses indicate seasonal and circadian variations, showing increased incidence during winter months and early morning hours [33,34].

Chest pain is the primary clinical manifestation observed in patients with AAS [2,35,36,37]. The pain typically presents abruptly, is severe and sharp, and is often described as tearing, frequently radiating along the anatomical pathway of the affected aortic segment and its branches. Type A AAS typically presents with pain localised to the anterior chest, while type B AAS is more frequently associated with posterior thoracic or interscapular discomfort. Painless presentations are infrequent.

Physical investigation may demonstrate specific observations, such as an early diastolic murmur indicative of aortic regurgitation, pulse deficits, or asymmetric peripheral pulses. AAS must be evaluated in patients exhibiting profound hypotension, syncope, or circulatory shock, especially when these symptoms are associated with chest pain. This diagnostic mode is endorsed by Class I recommendations [2,27]. The existence of several predisposing risk factors increases clinical suspicion and requires prompt diagnostic assessment.

The symptomatology of AAS can resemble other cardiovascular or oesophageal disorders, highlighting the necessity for a thorough diagnostic evaluation. Imaging modalities, especially contrast-enhanced computed tomography (CT), are fundamental to diagnosis, supported by chest radiography, electrocardiography, and laboratory biomarkers. Management strategies include aggressive pharmacologic therapy for haemodynamic stabilisation and, in many instances, timely surgical intervention. The prognosis is significantly influenced by the prompt identification and timely commencement of definitive treatment.

Smoking contributes to vascular inflammation, atherosclerosis, COPD, impaired tissue oxygenation, and postoperative pulmonary complications. Although direct AAS cost analyses stratified by smoking status are limited, smoking-related comorbidity plausibly increases mechanical ventilation time, ICU stay, readmission risk, and rehabilitation needs. The manuscript now distinguishes between epidemiological association, mechanistic plausibility, and direct cost evidence. Cigarette smoking is a significant modifiable factor influencing cardiovascular morbidity and mortality, with established effects on the occurrence of coronary artery disease, cerebrovascular incidents, congestive heart failure, atrial fibrillation, sudden cardiac death, and the development of abdominal aortic aneurysms [38,39]. Recent epidemiological studies suggest that active tobacco use may be a risk factor for aortic dissection [9,40,41,42,43,44]. However, the existing literature is constrained by a lack of large-scale prospective cohort studies. Quantitative analyses demonstrate that former smokers show a relative risk reduction of about 48–75% in comparison with present smokers. Moreover, prolonged smoking cessation is linked to a reduced risk in a graded manner, as shown in longitudinal studies [41]. There is a documented dose–response relationship indicating that higher tobacco exposure, as quantified by daily cigarette consumption and cumulative pack-years, is positively associated with the incidence of aortic dissection. An inverse dose–response relationship has been observed between years since cessation and residual risk, highlighting the potential for risk reduction through prolonged abstinence. The findings indicate a biologically plausible connection between chronic tobacco exposure and aortic wall integrity, necessitating further mechanistic investigation and high-quality prospective studies to clarify causality and assess related risk.

## 5. Impact

### 5.1. Hospital Stays

Prolonged hospitalisation in AAS is driven by the interaction between preoperative status, anatomical extent, urgency of intervention, operative complexity, and postoperative complications. Patients with type A AAD frequently require emergency open repair and ICU care; patients with complicated type B disease may require TEVAR, hybrid strategies, or prolonged medical stabilisation. In both settings, malperfusion, renal dysfunction, respiratory failure, neurological injury, infection, and low cardiac output syndrome are major drivers of extended stay.

The complicated nature of AAS and the longer duration of surgical intervention demand extensive postoperative management in the intensive care unit (ICU), frequently surpassing that needed for standard cardiac or aortic procedures [45,46]. Clinical evidence suggests that advanced age and female gender serve as independent predictors of adverse outcomes, leading to increased ICU bed occupancy and resource utilisation. Elderly patients demonstrate increased vulnerability to severe postoperative complications, whereas female patients often exhibit atypical or less specific symptoms, leading to diagnostic delays and postponed surgical procedures [47].

Postoperative recovery exhibits notable disparities related to age. Patients aged 80 years and older experience considerably more hospitalisations and ICU stays in comparison with younger cohorts. A study conducted in Japan indicated that older patients with Type A AAD experienced mean hospital stays of 42.2 days, compared to 35.8 days for younger patients [48]. Advanced age significantly influences postoperative morbidity and mortality after surgical correction of Type A dissection. Complications, including multi-organ dysfunction, low cardiac output syndrome (LCOS), and nosocomial infections, lead to prolonged hospitalisation and heightened healthcare costs [49]. Elderly patients frequently exhibit diminished clinical manifestations, resulting in delays in diagnosis and surgical intervention. Recent data suggests that patients older than ≥70 have suffered notable delays from initial presentation to surgery, regardless of evidence suggesting that surgical repair is superior to medical therapy in appropriately selected candidates [50].

Data from the IRAD indicate that female patients with aortic dissection (AD) demonstrate distinctive clinical manifestations relative to males, involving a late hospital presentation and severe physiological compromise at admission, which includes coma and cardiac tamponade [51]. The reported greater mortality among women across multiple cohorts may be attributed in part to these characteristics, despite age adjustment, and to their higher probability of prehospital death compared to men [8,11,51]. In medically managed Type B dissections, female patients exhibit higher mortality rates while experiencing lower overall complication rates. In contrast, meta-analytic findings indicate no significant differences in early mortality based on gender after surgical repair of Type A dissections. Women with AAS often exhibit a greater prevalence of prior hypertension and atherosclerotic disease. The patient experiences significant inpatient complications, such as cardiac tamponade, hypoxaemia, and myocardial ischaemia, leading to prolonged hospitalisation and increased resource utilisation. Male patients diagnosed with Type A dissection exhibit a predisposition to complications, including acute renal failure and the need for reoperation, which frequently require intricate surgical interventions such as aortic root replacement [52,53,54,55].

Pre-existing hypertension is identified as a notable risk factor for postoperative LCOS, likely indicative of chronic myocardial strain [56]. Hypertension is common in patients who undergo cardiac surgery that necessitates cardiopulmonary bypass (CPB) [57]. CPB duration and postoperative LCOS are significant predictors of extended ICU stay after surgical correction of acute Type A dissection. Michalopoulos et al. (1996) showed that prolonged CPB time significantly affects ICU length of stay, with multivariate analyses indicating that CPB duration is a more influential factor than aortic cross-clamp time [58]. In this population, advanced age significantly increases the risk of prolonged ICU stay and perioperative mortality.

The early detection of Loeys–Dietz syndrome before aortic surgery correlates with enhanced clinical outcomes, such as a decreased frequency of emergency procedures, reduced necessity for arch reconstruction, shorter hospital stays, and a lower likelihood of subsequent aortic reintervention. Moreover, prompt diagnosis aids in the preservation of the native aortic valve and reduces the need for long-term anticoagulation, especially in younger patients [59].

A substantial percentage of individuals (30–50%) with Stanford type A aortic dissection die before reaching the hospital, either at home or en route [8,11,60]. Estimating hospital-related costs based solely on epidemiological data presents significant challenges. The incidence of clinically recognised AAS is on the rise. The evaluation of the largest national database in the U.S. indicates an increase in hospital admissions for AD from 2012 to 2016, with in-hospital mortality consistently high at around 26.0% throughout this timeframe [20].

### 5.2. Bed Occupancy

Genetic disorders, including Marfan syndrome, Loeys–Dietz syndrome, and vascular Ehlers-Danlos syndrome (vEDS), significantly impact bed occupancy for AAS by increasing the incidence, complexity, and length of hospitalisations. Patients with such illnesses require extensive surveillance and often need emergency or speciality surgical interventions, resulting in a substantial and prolonged reliance on critical care and operating room resources. Those diagnosed with Marfan Syndrome exhibit higher bed occupancy rates, as it is the most common inherited aortopathy. The substantial risk of aortic root dilation requires prophylactic surgical intervention. Aortic root replacement is commonly conducted in both elective and emergency settings. Requires ongoing monitoring of the aorta throughout an individual’s lifespan. Patients with Loeys–Dietz Syndrome exhibit elevated bed occupancy rates due to the aggressive and extensive aortopathy associated with the condition. May lead to dissections at smaller aortic diameters and at a younger age. Furthermore, it is associated with a higher frequency of emergency surgeries and repeat procedures, particularly when diagnosis is delayed [61].

### 5.3. Hospital Outcome

Short- and intermediate-term mortality are strongly influenced by haemodynamic compromise, advanced age, renal dysfunction, need for cardiopulmonary resuscitation, tamponade, malperfusion, and operative complexity. These variables also affect resource use because patients who survive the acute phase often require prolonged ICU support, additional procedures, renal replacement therapy, neurological care, or complex rehabilitation.

For elderly patients, contemporary outcome studies show that chronological age alone should not be used as an absolute contraindication to intervention. Instead, frailty, malperfusion, preoperative neurological status, renal function, institutional experience, and time to surgery should inform the decision. This nuance was added to avoid overgeneralising from single-country or single-centre datasets.

A multicentre cohort study assessed the corresponding impacts of clinical and temporal covariates on short- and intermediate-term mortality after acute AAD. A graded dose–response relationship was identified between reduced admission systolic blood pressure (SBP) and increased all-cause mortality rates during hospitalisation, as well as at 30 days and 6 months post-admission. Lower admission SBP was identified as an independent predictor of mortality following multivariable adjustment, including logistic regression for in-hospital and 30-day outcomes, as well as Cox proportional hazards modelling for 6-month outcomes. This finding aligns with the notion that haemodynamic compromise is a critical prognostic factor. Increased age was similarly linked to a gradual decline in short- and intermediate-term survival [62]. The age effect is likely mediated by an increased burden of multimorbidity and mortality [63], encompassing comorbid conditions associated with negative post-AAD outcomes [64].

Findings from the surgical literature identify significant prognostic factors for early mortality following operative repair of acute type A AAD. Both univariate and multivariable analyses indicate that advanced age, preoperative renal dysfunction, preoperative haemodynamic instability, the need for cardiopulmonary resuscitation (CPR), cardiac tamponade, the omission of retrograde cerebral perfusion (RCP) during arch reconstruction, and prolonged deep hypothermic circulatory arrest (DHCA) duration are consistently linked to an increased mortality risk, maintaining importance after adjusting for confounding variables [45,46,56,57,65,66,67,68].

### 5.4. Cost

The economic impact of AAS extends beyond the index admission. Initial costs arise from emergency diagnostics, transfer to aortic centres, ICU stay, complex open or endovascular repair, blood products, mechanical ventilation, and management of complications. Post-discharge costs include readmissions, rehabilitation, imaging surveillance, antihypertensive therapy, genetic counselling/testing in selected families, and reintervention for distal aortic remodelling or graft-related complications.

Cost interpretation should distinguish between median per-admission cost and aggregate system-wide cost. Earlier diagnosis may increase detection of medically managed or smaller aortic pathology, potentially reducing median per-patient procedural cost while increasing total case volume and longitudinal surveillance expenditure. Consequently, “lower median cost” should not automatically be interpreted as reduced health-system burden.

Everyone with hypertension requires continuous antihypertensive therapy throughout their lives. β-adrenergic receptor antagonists (β-blockers) are the primary pharmacologic treatment, aiming for a target arterial pressure of <120/80 mmHg in the majority of cases [69,70,71]. Regular imaging surveillance of the thoracic aorta is advised at 1, 3, 6, 9, and 12 months following discharge, with subsequent intervals of 6 to 12 months based on aortic dimensions [72]. Key radiologic parameters encompass maximal aortic diameter, signs of aneurysmal remodelling, and bleeding complications at surgical anastomotic sites or endovascular stent-graft interfaces. This comprehensive follow-up strategy highlights the clinical observation that systemic hypertension and progressive aortic dilation/dissection are common and unanticipated in the early postoperative period. Chronic hypertension and advancing aortic conditions require extended pharmacological treatment, potentially leading to considerable financial implications.

Direct cost studies for AAS categorised by smoking status are scarce; however, projections from more general cardiovascular and aneurysm-related data sources suggest that smokers show higher resource utilisation. Patients demonstrate increased hospitalisation rates and longer periods in the intensive care unit (ICU), due to intricate surgical procedures and perioperative complications. In smokers with advanced aortic disease, there is an increased dependence on mechanical ventilation, vasoactive agents, and circulatory support, which further raises costs. Moreover, comorbidities associated with smoking, including COPD and peripheral arterial disease, lead to prolonged hospital stays and increased readmission rates, thereby escalating healthcare costs. Tobacco-related diseases significantly burden healthcare systems, with smoking being a primary contributor to acute care expenditures [73]. After hospitalisation for acute aortic dissection or thoracic aortic aneurysm, a greater percentage of female patients, in contrast to male patients, engage with post-discharge medical facilities during the rehabilitation period. Gender-specific differences in the management of aortic dissection persist past perioperative care and greatly impact long-term recovery trajectories. The emergency nature of type A dissections requires immediate referral to tertiary care centres that have advanced cardiovascular surgical expertise upon diagnosis, leading to increased healthcare costs. Hospital readmissions within 12 months post-discharge are prevalent in this cohort, with secondary interventions during these episodes incurring the highest median cost for commonly utilised post-hospitalisation services [74].

Identifying risk factors prior to the initial phase may aid in reducing expenses. While various factors affect these patterns, specific conclusions can be made. A reason for the reported reductions in average costs is the initial identification of aortic pathology, facilitated by improvements in diagnostic imaging techniques. An accurate diagnosis enhances the detection of small aneurysms that fall below the operative threshold, thereby promoting conservative medical management instead of surgical intervention. The increase in the medically managed subgroup compared to surgically treated cases results in reduced median hospitalisation costs for the overall aortic dissection population. This may lead to a perceived decrease in costs that is, in truth, a statistical artefact indicating lower median expenditures amid a higher incidence of disease, ultimately causing an increase in overall system-wide costs [74]. Reoperation is required in about 12% to 30% of patients, mainly due to the advancement or relapse of aortic dissection at the previous intervention site, the development of aneurysmal dilation distal to the original repair, prosthetic graft dehiscence, clinically significant aortic regurgitation, or infectious complications [75,76].

Dedicated aortic pathways and regional networks may reduce delays, improve triage, and concentrate expertise. Their value should be evaluated not only by mortality but also by time-to-diagnosis, time-to-surgery, transfer efficiency, ICU days, reintervention, readmission, and total cost of care.

## 6. Practical Implications for Risk Stratification and Hospital Planning

The narrative review supports a two-axis approach to hospital planning: (1) clinical risk, including subtype, anatomy, malperfusion, haemodynamics, age/frailty, sex-related diagnostic risk, and genetic background; and (2) resource risk, including likelihood of ICU admission, long operative time, transfusion, prolonged ventilation, renal replacement therapy, rehabilitation, readmission, and lifelong surveillance. This approach may help hospitals identify patients who need early transfer, specialist aortic-team involvement, and proactive ICU/operating-room allocation.

Future studies should report AAS outcomes using harmonized endpoints: ICU length of stay, total hospital length of stay, ventilator days, ward bed days, time to diagnosis, time to definitive treatment, reoperation, readmission, rehabilitation destination, and cost. These endpoints would allow stronger comparisons across healthcare systems and clarify which interventions reduce preventable resource use.

## 7. Limitations

This review is narrative and therefore subject to selection bias. It does not provide pooled estimates, formal risk-of-bias assessment, or certainty grading. Many included studies are retrospective, registry-based, or single-centre, and they use heterogeneous definitions of prolonged LOS, ICU stay, complications, and cost. Findings derived from administrative databases may be influenced by coding practices and discharge policies. These limitations are now stated explicitly to align the claims with the level of evidence.

## 8. Conclusions

AAS generates a high hospital resource burden because its clinical course is shaped by time-critical diagnosis, anatomical complexity, haemodynamic instability, malperfusion, comorbidity, and the need for specialised intervention. Hypertension, advanced age, sex-related diagnostic patterns, smoking-related comorbidity, hereditary aortopathy, and operative complexity are not only risk factors for disease or mortality; they also influence ICU occupancy, total hospital stay, readmission, reintervention, and long-term surveillance costs.

The main contribution of this revised review is a resource-oriented synthesis that links risk factors to hospitalisation duration and cost. Early recognition of high-risk presentations, standardised diagnostic algorithms, dedicated aortic pathways, genetic evaluation in appropriate patients, smoking cessation, hypertension control, and cautious use of medications associated with aortic risk may reduce preventable morbidity and unnecessary resource use.

Crucially, disparities in risk classification and delayed diagnosis across healthcare systems lead to needless morbidity, mortality, and resource waste. ICU reliance, unexpected presentations, and catastrophic complications can be decreased by using improved clinical algorithms, genetic screening in certain groups, and sophisticated imaging to detect high-risk patients. The occurrence and severity of sickness can be reduced by aggressively managing modifiable risk factors, particularly smoking cessation, hypertension control, and the cautious use of potentially harmful medications such as fluoroquinolones.

For health systems, AAS is a clinical and financial issue. Direct and indirect expenditures are increased by lengthy hospitalisations in the intensive care unit, challenging procedures, readmissions, and lifetime monitoring. Future research should prioritise multicentre, prospectively collected datasets with harmonised LOS, ICU, cost, and patient-centred recovery endpoints. Predictive models, including machine-learning approaches, should be externally validated and evaluated for clinical utility before routine implementation.

## Figures and Tables

**Table 1:** Review strategy.

Element	Description
Databases	PubMed/MEDLINE; Scopus; Web of Science; Google Scholar
Time window	January 2000–May 2026, with emphasis on 2021–2026 literature
Core concepts	AAS entities; patient risk factors; operative complexity; ICU stay; hospital LOS; bed occupancy; cost
Inclusion focus	Guidelines, registries, population cohorts, observational studies, systematic reviews, cost/resource-use studies
Interpretation	Narrative synthesis; no pooled meta-analysis; study design and generalisability considered

**Table 2:** Narrative strategy synthesis.

Risk Factor	Evidence Base	Link to Adverse Clinical Course	Likely Resource Consequence
Hypertension	Population cohorts, registries, guidelines	Increased AAS risk; chronic aortic remodelling; acute haemodynamic instability	ICU blood pressure titration, organ-protection monitoring, lifelong therapy, and imaging
Advanced age	Administrative cohorts, surgical series, contemporary elderly cohorts	Higher comorbidity burden, delayed diagnosis, reduced physiological reserve	Longer ICU and ward recovery, rehabilitation needs, discharge delays
Female sex	Registry analyses, meta-analyses, sex-specific cohorts	Older presentation, atypical symptoms, delayed recognition, tamponade/malperfusion risk	Higher acuity at admission, increased diagnostic and postoperative resource use
Smoking/COPD	Prospective cohorts, case–control studies, mechanistic evidence	Atherosclerosis, pulmonary complications, impaired recovery	Ventilation, ICU dependency, readmission, rehabilitation costs
Hereditary aortopathy	Guidelines, genetic/aortopathy reviews, surgical cohorts	Earlier onset, complex anatomy, recurrent disease, need for staged repair	Specialised surgery, genetic testing, lifelong surveillance, reintervention
Malperfusion/hemodynamic compromise	Multicentre cohorts, surgical series	Renal, cerebral, coronary, mesenteric or limb ischaemia; low SBP/shock	Emergency surgery, organ support, prolonged ICU, mortality risk
Operative complexity/CPB time	Surgical observational studies, ICU-LOS models	Arch involvement, prolonged circulatory arrest, bleeding, coagulopathy	Longer operative and ICU time, transfusion, infection, LCOS
Delayed diagnosis/system factors	IRAD analyses, emergency pathway studies, aortic network literature	Late transfer, advanced complications, missed atypical presentations	Higher emergency burden, avoidable ICU days, increased cost
Biomarker/clinical prediction variables	Recent machine-learning LOS studies	SBP, operative time, HDL-C, ALT and lymphocyte percentage associated with prolonged LOS in a single-centre AD cohort	Potential preoperative risk stratification; requires external validation

## Data Availability

The data supporting this study’s findings are available from the corresponding author upon reasonable request.
